# Research progress and hotspot of the artificial intelligence application in the ultrasound during 2011–2021: A bibliometric analysis

**DOI:** 10.3389/fpubh.2022.990708

**Published:** 2022-09-15

**Authors:** Demeng Xia, Gaoqi Chen, Kaiwen Wu, Mengxin Yu, Zhentao Zhang, Yixian Lu, Lisha Xu, Yin Wang

**Affiliations:** ^1^Luodian Clinical Drug Research Center, Shanghai Baoshan Luodian Hospital, Shanghai University, Shanghai, China; ^2^Department of Pancreatic Hepatobiliary Surgery, Changhai Hospital, Naval Medical University, Shanghai, China; ^3^Department of Gastroenterology, The Third People's Hospital of Chengdu, The Affiliated Hospital of Southwest Jiaotong University, Chengdu, China; ^4^Department of Ultrasound, Shanghai Pulmonary Hospital, Tongji University School of Medicine, Shanghai, China; ^5^Department of Clinical Medicine, The Naval Medical University, Shanghai, China

**Keywords:** bibliometrics, artificial intelligence, ultrasound, CNN, COVID-19

## Abstract

Ultrasound, as a common clinical examination tool, inevitably has human errors due to the limitations of manual operation. Artificial intelligence is an advanced computer program that can solve this problem. Therefore, the relevant literature on the application of artificial intelligence in the ultrasonic field from 2011 to 2021 was screened by authors from the Web of Science Core Collection, which aims to summarize the trend of artificial intelligence application in the field of ultrasound, meanwhile, visualize and predict research hotspots. A total of 908 publications were included in the study. Overall, the number of global publications is on the rise, and studies on the application of artificial intelligence in the field of ultrasound continue to increase. China has made the largest contribution in this field. In terms of institutions, Fudan University has the most number of publications. Recently, IEEE Access is the most published journal. Suri J. S. published most of the articles and had the highest number of citations in this field (29 articles). It's worth noting that, convolutional neural networks (CNN), as a kind of deep learning algorithm, was considered to bring better image analysis and processing ability in recent most-cited articles. According to the analysis of keywords, the latest keyword is “COVID-19” (2020.8). The co-occurrence analysis of keywords by VOSviewer visually presented four clusters which consisted of “deep learning,” “machine learning,” “application in the field of visceral organs,” and “application in the field of cardiovascular”. The latest hot words of these clusters were “COVID-19; neural-network; hepatocellular carcinoma; atherosclerotic plaques”. This study reveals the importance of multi-institutional and multi-field collaboration in promoting research progress.

## Introduction

Ultrasound is a tool capable of visualizing the structure of organs including thyroid, breast, liver and cardiovascular, which makes ultrasound widely applied in the diagnosis of diseases and monitoring the pathology or physiology changes. As a common inspection method of modern clinical medicine, ultrasound has advantages over CT and MRI by being cheap, portable and the capability of real-time imaging (1, 2). For example, during the diagnosis of COVID-19, pulmonary ultrasound is of more and more importance in the clinical decision-making as a quick and accurate way of diagnosis, especially in the management of the patients with lung injury or respiratory failure.

Recently, the emergence of artificial intelligence (AI) and its application in the field of medicine provides a potential solution to relevant bottlenecks. AI is an advanced computer program with a specific goal, which can present different responses based on changes of parameters that it receives or collects to achieve an established target (3). Machine learning (ML) is a research method of artificial intelligence, while DL is an important algorithm of machine learning. And DL, one of the algorithms of machine learning, helps computers learn and analyze by building the Artificial Neural Networks, which is a model of human neurons for layer-by-layer transmission of data processing (4). DL is characterized by the use of multi-layer neural networks for data processing, so it is more conducive to image recognition and processing of complex data. Through deep learning, artificial intelligence can identify images and analyze the data from them (5). DL enables the AI to quantitative analyze the image information of the ultrasound image which includes quantifying the image data into digital data, presenting the invisible subtle differences in a more accurate and swift way by training machine learning with massive repeating data, cutting medical costs and optimizing clinical processes (3). Therefore, AI has developed rapidly in medical imaging in the liver, breast and kidney, and hundreds of articles have been published by scholars around the world (6–8).

Although AI has made much progress in the application of ultrasound, this field still lacks a global and comprehensive report to help researchers quickly understand the research trend and hotspots of this area. Bibliometrics, a research method for quantitative analysis of literature, can make statistical analysis of research in current popular fields by applying mathematics and statistics to obtain valuable information and predict the direction of research in next stage according to the data (9). Through the visual chart, bibliometrics intuitively display of the results of the analysis, and highlighting the impact of existing research on the subject. Bibliometrics has been widely used in the medicine to help researchers to identify more specific research topics, thus more intuitive understanding of the relationship between the various fields.

**Figure 1:**
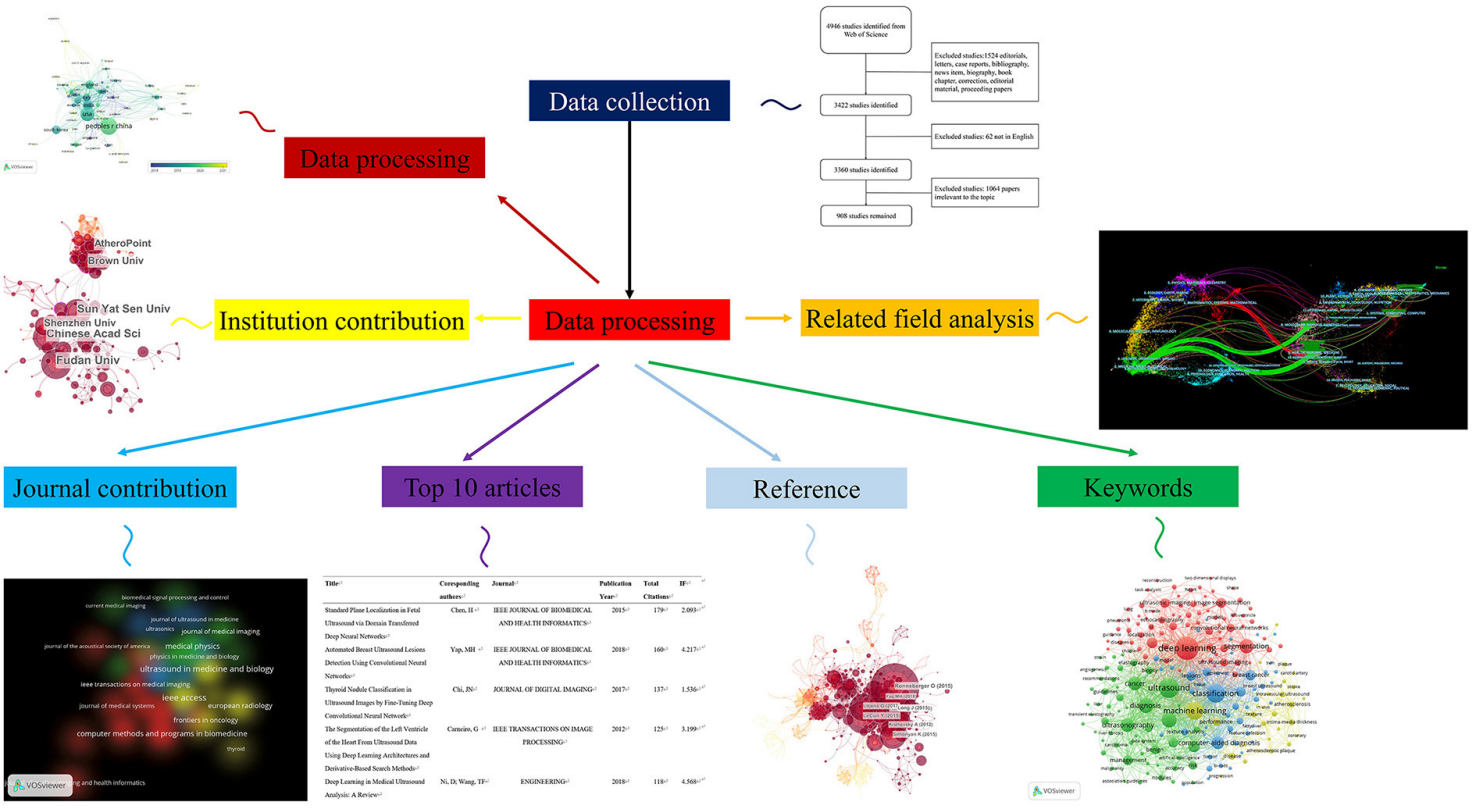
Flow chart of artificial intelligence application in ultrasonic field in five aspects. List the contributions of countries, different institutions, different journals and the top 10 articles, keywords, and related fields.

The purpose of this paper is to comprehensively analyze the relevant literature in the field from 2011 to 2021 in the core collection of the Web of Science (WOS) database, analyze and demonstrate the research trends in this field, and predict the future research hotspots. As shown in Figure 1, we studied and discussed the publications, citations, keywords and publication trends of “AI technology in the field of ultrasound” through the method of bibliometrics for the first time. After that, we summarized and analyzed 5 ongoing clinical trials. And combining the research contents of clinical trials with the current hot issues, we expound the relationship between theoretical research and clinical application, and put forward the necessity and scientificity of the transformation from theory to practice.

## Methods

### Data sources and search strategies

Based on the Web of Science Core Collection database, we conducted a comprehensive search of literature on ultrasonic artificial intelligence from 2011 to 2021. The search strategy is as follows: TS = (ultrasound OR ultrasonography OR ultrasonic OR sonography) and (Artificial intelligence OR Machine intelligence OR artificial neutral network OR Machine learning OR Deep learn^*^ OR Natural language process^*^ OR Robotic^*^ OR thinking computer system OR fuzzy expert system^*^ OR evolutionary computation OR hybrid intelligent system^*^). We qualified English as the only language and exclude editorials, letters, case reports, bibliography, news item, biography, book chapter, correction, editorial material and proceeding papers. In order to avoid deviation caused by database update, all literature retrieval was completed on August 8, 2021. Two authors (DMX and GQC) extracted title, author, key words, abstract, references and other information from all qualified literatures, and exported them in TXT format.

All the data (title, keywords, author, country and region, publisher, date of publication, H-index, total citations, etc.) are all from the WOS database. The following tools are used to present, analyze and describe the data: Microsoft Excel, GraphPad Prism 8, VOSviewer (a program operated by the Center for Science and Technology Studies at Leiden University, were used to create data maps), and CiteSpace (from 5.7.R2 64-bit, Chaomei Chen, Drexel University, USA).

All clinical trial data were derived from Clinicaltrail Database (https://www.clinicaltrials.gov/), and “Artificial intelligence” and “ultrasound” were used as keywords for retrieval. We through manual screening obtained 5 clinical trial reports, and the detail information about the report was concluded.

### Bibliometric analysis

The study collected a large amount of data from WOS, especially the data concerning AI and ultrasound. The H-index indicates that a researcher or a country has published at most H papers, and each paper has been cited by other publications at least H times. It is a practical indicator for assessing scientific achievement. Relative research interest (RRI) is defined as the number of publications in a specific area divided by the total number of publications per year. Impact factor (IF) is provided by Journal Citation Reports (JCRS). Recently, H-Index, RRI and IF have been used to assess a scientific research impact of a researcher or a country. Vosviewer, a scientific drawing software, which is based on a commonly built-in construction and view author, journal or keyword documentary, is applied to the analysis of countries, authors and keywords in our research. Citespace is a Java application for analyzing and visualizing co-citation networks, its main goal is to promote the analysis of developing fields. It can be used for summarizing keyword clusters and overall researching directions.

## Results

**Figure 2:**
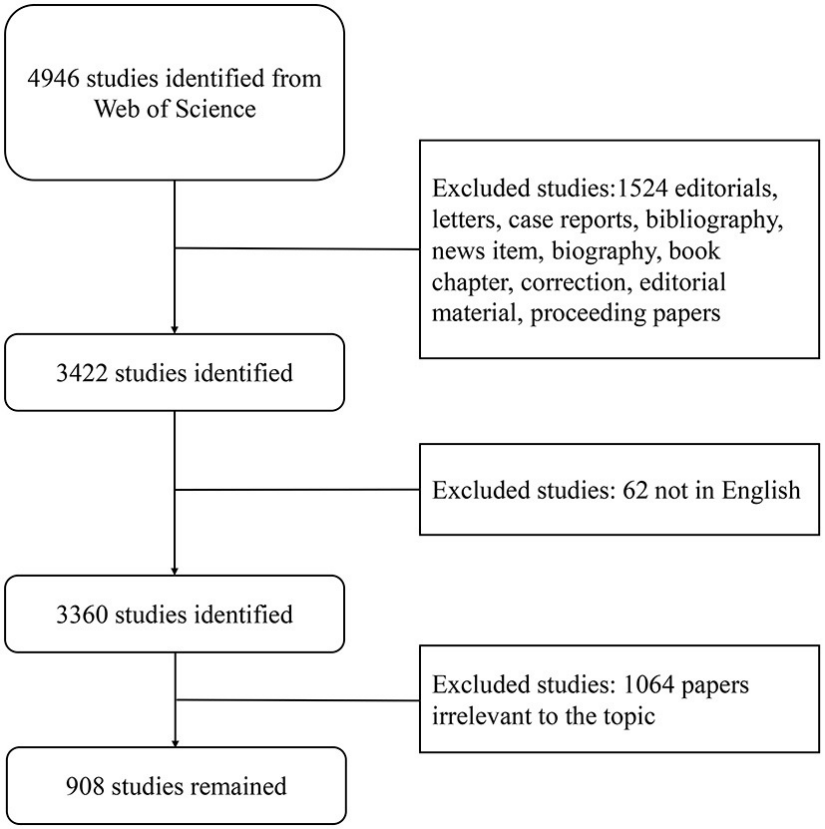
Flow chart of literature screening. The detailed process of filtering (by two authors manually filtering irrelevant articles through abstract and full text, excluding irrelevant articles).

This research analyzed 908 publications (1,524 articles were excluded because of editorials, letters, case reports, bibliographies, news, biographies, bibliographic chapters, revisions, editorial materials, and proceeding papers which did not fit the stylistic type of our analysis. Sixty-two non-English papers and 1,064 that did not meet the requirements were excluded through manual screening. Finally, 908 studies that passed the screening were retained and analyzed) (Figure 2) focusing on AI in ultrasound from the whole world between 2011 and 2021.

### Contribution of countries and regions to global publication volume

In the figures of the visual analysis with Contribution, this study shows the distribution of publications in different countries and regions, and the results include the total number of publications and their popularity. We chose the country of the first corresponding author as the country of this publication. The top countries include China, the U.S., India and South Korea. Since 2011, China has published 317 publications in this field, accounting for 34.91% of the total. It was followed by the United States with 208 articles (22.91%) while India was the third with 85 articles (9.36%) (Table 1).

**Table 1:** Top 20 countries with the highest global contributions related to the use of AI in ultrasound.

**Country**	**No. of** **publications**	**Sum of** **citations**	**Citations**	**H-index**
Peoples R China	317	2,313	1,981	26
USA	208	1,973	1,763	25
India	85	781	643	17
South Korea	79	941	863	18
England	78	919	828	17
Canada	67	675	637	14
Italy	60	826	693	17
Japan	51	472	427	13
Spain	35	702	680	13
Germany	28	234	228	8
Taiwan	28	150	148	7
France	26	381	374	8
Netherlands	22	197	191	7
Cyprus	21	294	217	10
Australia	20	328	320	7
Poland	20	173	158	7
Greece	18	146	122	7
Switzerland	15	112	110	3
Norway	13	250	246	5
Singapore	13	197	195	5

According to the database of WOS, articles from the top 20 countries with the most publications have been totally cited 12,064 times since 2011 (10,824 times without self-citation), with an average citation frequency of 13.29 times per article. China accounted for 19.17% of the total citations, that is 2,313 citations (1,981 without self-citation), with an H index of 26. The United States and Korea, respectively, ranked second and third with 1,973 citations (1,763 without self-citation) and 941 citations (863 without self-citation). Further analysis by Vosviewer suggests that the United States has been working very closely with other countries.

**Figure 3:**
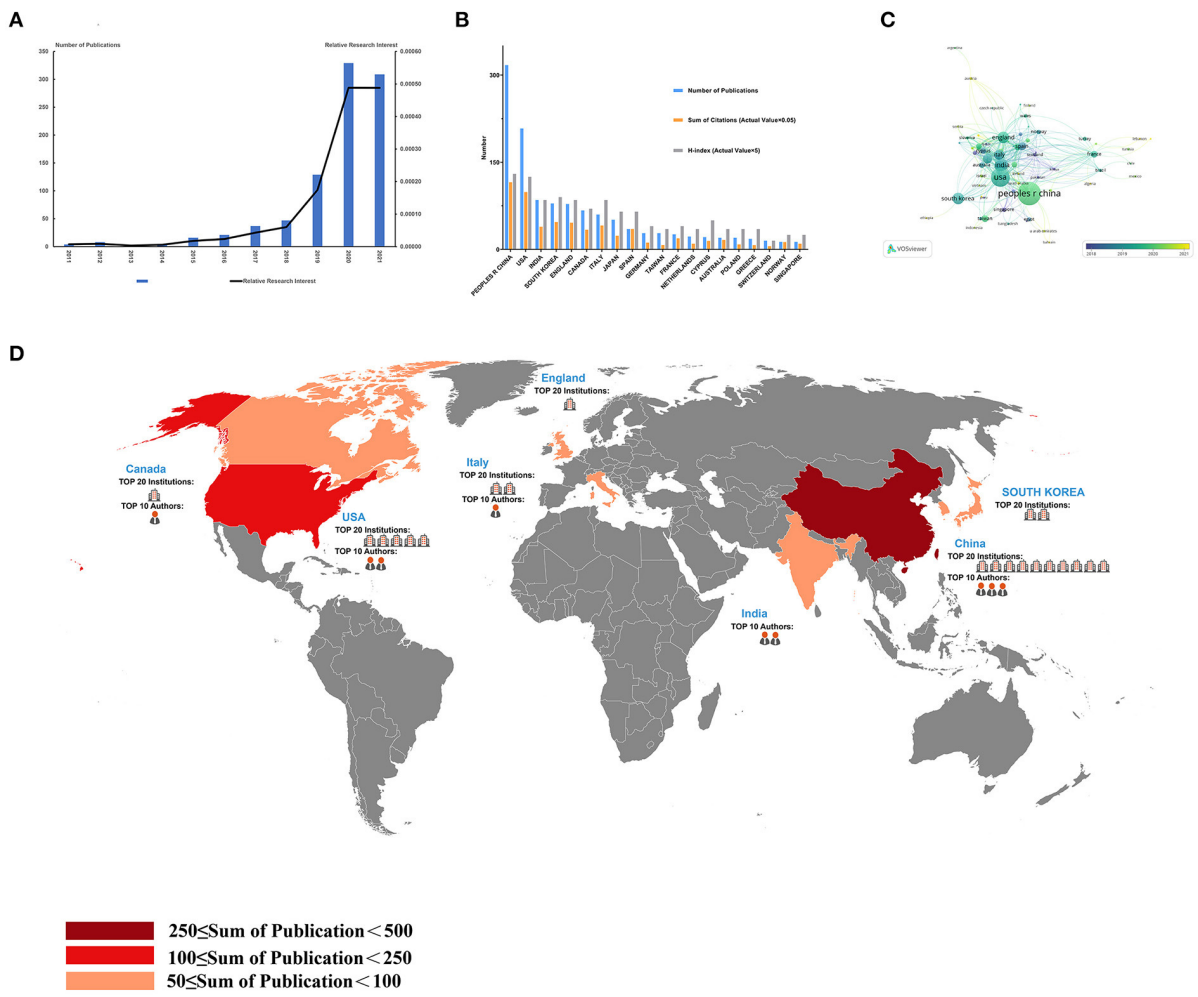
Contributions of different countries/regions to the application of artificial intelligence in the field of ultrasound, and a global cooperation network in this field. **(A)** The world publication of RRI in the time course of artificial intelligence applications in the field of ultrasound; **(B)** Total publications, total citations and H-index of the 20 most productive countries/regions; **(C)** A network of cooperative relations between States; **(D)** country contributions, institutions and the distribution of the Top 10 authors.

As shown in Figure 3, by studying the annual issuance, it was found that the global total number of articles published in last decade shows a continuous increasing trend. 2020 is the year with the largest number of articles published, which witnessed 329 articles being published (36.23%) (Figure 3A). Compared with publications in all fields, global research hotspots measured by the RRI index rise exponentially. Since we could only collect the data from January to August in 2021, we were not able to infer that the number of publications would keep rising in 2021, but we can predict that the development of the research in this field will not stop accelerating in the future.

### Contribution of different institutions to publication volume

Through the visual analysis of the research results by VOSview, we display the global institutions of the number of publications and the publications of each institution according to the time sequence. Among the global institutions, Fudan University had the largest number of papers, with 31 published papers, accounting for 3.41% of the total (Table 2). Among the top 20 institutions with the most publications in this field, 10 were from China and 5 were from the United States. The top 3 institutions were Fudan University, Hinese Academy of Sciences and Sun Yat sen University, two of which were from China.

**Table 2:** Top 20 institutions with most numbers of publications related to the use of AI in ultrasound.

**Institution**	**Country**	**No. of publications**	**No. of citations**
Fudan University	Peoples R China	31	265
Hinese Academy of Sciences	Peoples R China	29	524
Sun Yat Sen University	USA	26	225
Shenzhen University	Peoples R China	21	530
Atheropoint	USA	17	243
Brown University	USA	17	227
Huazhong University of Science Technology	Peoples R China	17	112
Peking University	Peoples R China	17	98
Shanghai Jiao Tong University	Peoples R China	17	98
Yonsei University	Korea	15	111
Shanghai University	Peoples R China	14	232
Eindhoven University of Technology	Netherlands	14	161
Imperial College London	England	13	114
Mayo Clinic	USA	13	49
VASC Screening Diagnost CTR	USA	13	239
Beihang University	Peoples R China	13	105
Queens University	Canada	12	72
Seoul National University	Korea	12	150
Zhejiang University	Peoples R China	12	63
Harbin Institute of Technology	Peoples R China	12	91

**Figure 4:**
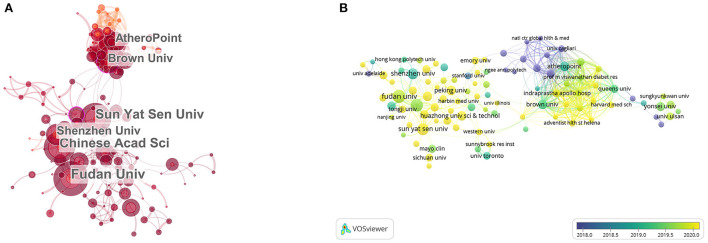
Distribution of publications on the application of artificial intelligence in the field of ultrasound in all institutions. **(A)** The network of institutions by Citesapce. **(B)** The average annual publication number network of institutions by VOSviewer. The size of the circle indicates the number of publications, green and blue circles indicate more past publications, and yellow circles indicate more recent publications.

As shown in Figure 4, circle size represents the number of publications published by this institution and the color from dark to light represents the publishing time of publications. Blue and green represent the early publishing time of literatures and yellow represents literatures published recently by this institution (Figure 4B). Among the top 20 institutions (Table 2; Figure 4B), Vasc Screening Diagnost Ctr published more publications in the past, while Imperial College London published more publications in recent years, indicating that the latter may achieve more in this field in the future.

### Contribution of different journals to publication volume

The results of the figures and tables show the publications of different journals in this field. Table 3 lists the top 20 journals in the number of publications in this certain field, the top 3 journals relatively were “IEEE Access” (IF =3.367), “Ultrasound In Medicine and Biology” (IF = 2.998) and “IEEE Transactions on UltraSonics Ferroelectrics and Frequency Control” (IF = 2.725). “IEEE Access” published the most articles (37 articles) in recent years, accounting for 9.56% of the top 20 journals with 144 citations which rank the 11th in the quantity of the citations. The second and third journals were “Ultrasound in Medicine and Biology” (32 articles) and “IEEE Transactions on UltraSonics Ferroelectrics and frequency control” (28 articles) (Figure 4). The remarkable phenomenon was that “IEEE Access” and “IEEE Transactions on UltraSonics Ferroelectrics and frequency control” were both from the IEEE Press.

**Table 3:** Top 20 journals with most numbers of publications related to the use of AI in ultrasound.

**Journal**	**No. of publications**	**Citations**
IEEE Access	37	144
Ultrasound IN Medicine and Biology	32	285
IEEE transactions on Ultrasonics Ferroelectrics and Frequency Control	28	103
Computer Methods and Programs in Biomedicine	24	256
Medical Physics	24	253
European Radiology	22	219
IEEE Journal of Biomedical and Health Informatics	21	553
International Journal of Computer Assisted Radiology and Surgery	20	149
Computers in Biology and Medicine	18	152
Frontiers in Oncology	18	46
Diagnostics	17	81
Sensors	17	31
Medical Image Analysis	16	153
Scientific Reports	16	73
IEEE Transactions on Medical Imaging	15	287
Journal of Medical Imaging	14	54
Journal of Digital Imaging	13	265
PLoS ONE	12	106
Ultrasonic Imaging	12	38
Physics in Medicine and Biology	11	129

Nine of the twenty journals with the most publications listed in Table 3 were founded in the United States. It is worth noting that IEEE, a scientific research institution in the United States, relates to journals including “IEEE Access” (IF = 3.367), “IEEE Transactions on Ultrasonics Ferroelectrics and frequency control” (IF = 2.725), “IEEE Journal of Biomedical and health information” (IF = 5.772), “IEEE Transactions On Medical Imaging” (IF = 10.048). Those four journals in the field of AI in ultrasound published 101 articles, accounting for 26.1% of the total number of papers published in the top 20 journals with the most publications. The total number of citations was 1,087, accounting for 32.2% of the total number of papers published in the top 20 journals with the most publications.

### Top 10 authors in the number of publications

The publication status of different authors in this field is shown by a visual figures and tables, from which we can get the results of Top 10 authors. As shown in Table 4, SURI JS published most of the articles in this field (29 articles). SURI JS also had the higher number of citations than any author (432 citations). Of the top 10 authors, two were from the US and three were from China. Two authors from the United States published 50 articles (27.6%) and their publications were cited 725 times (29.5%) in this field. Three authors from China published 45 articles (24.9%) and their publications were cited 669 times (27.2%). As we can infer from the specific data and proportion, the United States still had a leading position in the field of publications which were mainly published by AtheroPointTM and Adventist Hlth St Helena. In contrast, articles published by Chinese authors in this field were evenly distributed which led the author from China with most publications lost the advantages in the comparison with their colleagues from the United States. However, among the top 10 authors, the total number of articles published by American and Chinese authors and the number of citations was relatively close and the United States was only slightly ahead.

**Table 4:** Top 10 authors with most numbers of publications related to the use of AI in ultrasound.

**Author**	**Country**	**Affiliation**	**No. of publications**	**No. of citations**
Suri JS	USA	AtheroPointTM	29	432
Saba L	Italy	Azienda Osped Univ	27	414
Laird JR	USA	Adventist Hlth St Helena	21	293
Nicolaides A	Cyprus	Cyprus Cardiovasc Dis Educ Res Trust	20	260
Wang Y	Peoples R China	Shenzhen University	16	328
Wang YY	Peoples R China	Fudan University	15	93
Khanna NN	India	Indraprastha APOLLO Hosp	15	248
Zhang Q	Peoples R China	Shanghai University	14	248
Viswanathan V	India	4 West Mada Church St Royapuram	13	103
Johri AM	Canada	Queens University—Canada	11	42

### Top 10 most-cited articles

**Figure 5:**
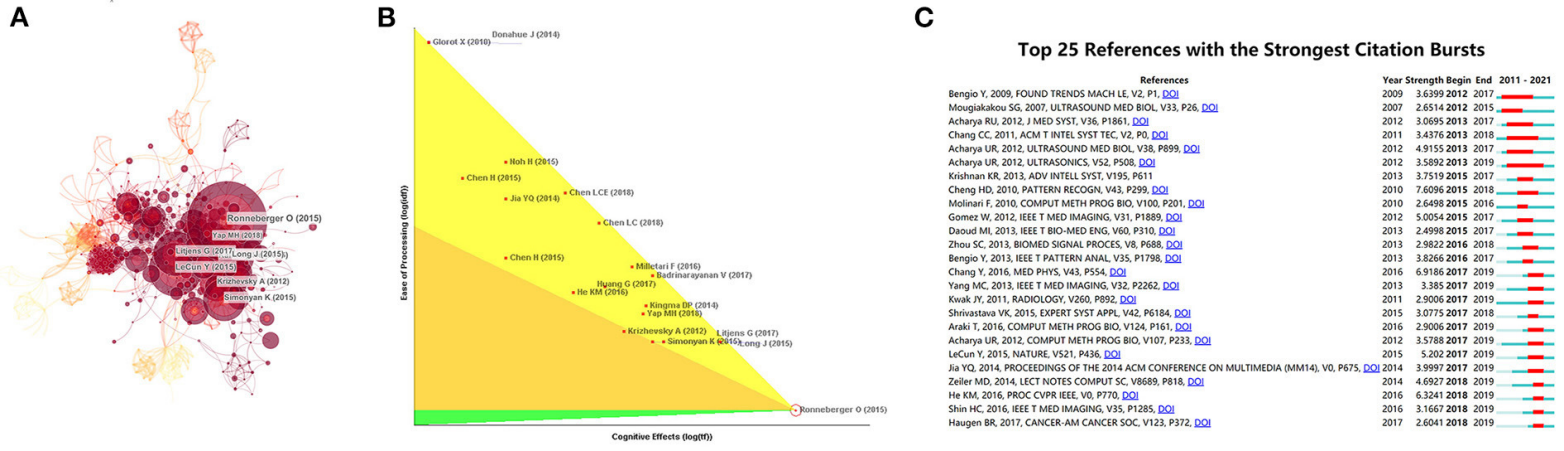
The network of references with the highest number of citations. **(A)** Clustering analysis of literatures co-citation network based on CiteSpace. **(B)** Article with the highest citation rate was processed in-depth analysis. **(C)** Top 25 references with strongest citation bursts based on CiteSpace.

Table 5 lists the top 10 most-cited articles. The most-cited article was “Standard Plane Localization in Fetal Ultrasound *via* Domain Transferred Deep Neural Networks” (IF = 2.093, 2015) which was cited 179 times. It was worth noting that the first and second most-cited publications were both published in IEEE Journal of Biomedical and Health Informatics. Co-citation, a research method to measure the relationship between articles, was initially proposed by an American intelligence scientist called Small in 1973. It can display articles with important influence in certain fields and use software Citespace to conduct simple co-citation analysis on documents (Figure 5A). Meanwhile, deep analysis was conducted on the literatures with the largest influence (Figure 5B) and top 25 references with the strongest citation surge (Figure 5C) can be obtained, showing that a certain article is being cited frequently at a certain stage, indicating that this article has a large contribution at this stage.

**Table 5:** Top 10 most-cited papers related to the use of AI in ultrasound.

**Title**	**Corresponding authors**	**Journal**	**Publication year**	**Total citations**	**IF**
Standard Plane Localization in Fetal Ultrasound *via* Domain Transferred Deep Neural Networks	Chen, H	IEEE Journal of Biomedical and Health Informatics	2015	179	2.093
Automated Breast Ultrasound Lesions Detection Using Convolutional Neural Networks	Yap, MH	IEEE Journal of Biomedical and Health Informatics	2018	160	4.217
Thyroid Nodule Classification in Ultrasound Images by Fine-Tuning Deep Convolutional Neural Network	Chi, JN	Journal of Digital Imaging	2017	137	1.536
The Segmentation of the Left Ventricle of the Heart From Ultrasound Data Using Deep Learning Architectures and Derivative-Based Search Methods	Carneiro, G	IEEE Transactions on Image Processing	2012	125	3.199
Deep Learning in Medical Ultrasound Analysis: A Review	Ni, D; Wang, TF	Engineering	2018	118	4.568
A deep learning framework for supporting the classification of breast lesions in ultrasound images	Seong, YK	Physics in Medicine and Biology	2017	111	2.665
Deep learning based classification of breast tumors with shear-wave elastography	Zhang, Q	Ultrasonics	2016	96	2.377
Efficacy of an Artificial Neural Network- Based Approach to Endoscopic Ultrasound Elastography in Diagnosis of Focal Pancreatic Masses	Gheonea, DI	Clinical Gastroenterology and Hepatology	2012	95	6.648
Transfer Learning with Convolutional Neural Networks for Classification of Abdominal Ultrasound Images	Cheng, PM	Journal of Digital Imaging	2017	94	1.536
Stacked deep polynomial network based representation learning for tumor classification with small ultrasound image dataset	Lu, MH	Neurocomputing	2016	94	3.317

### Co-occurrence keywords analysis

**Figure 6:**
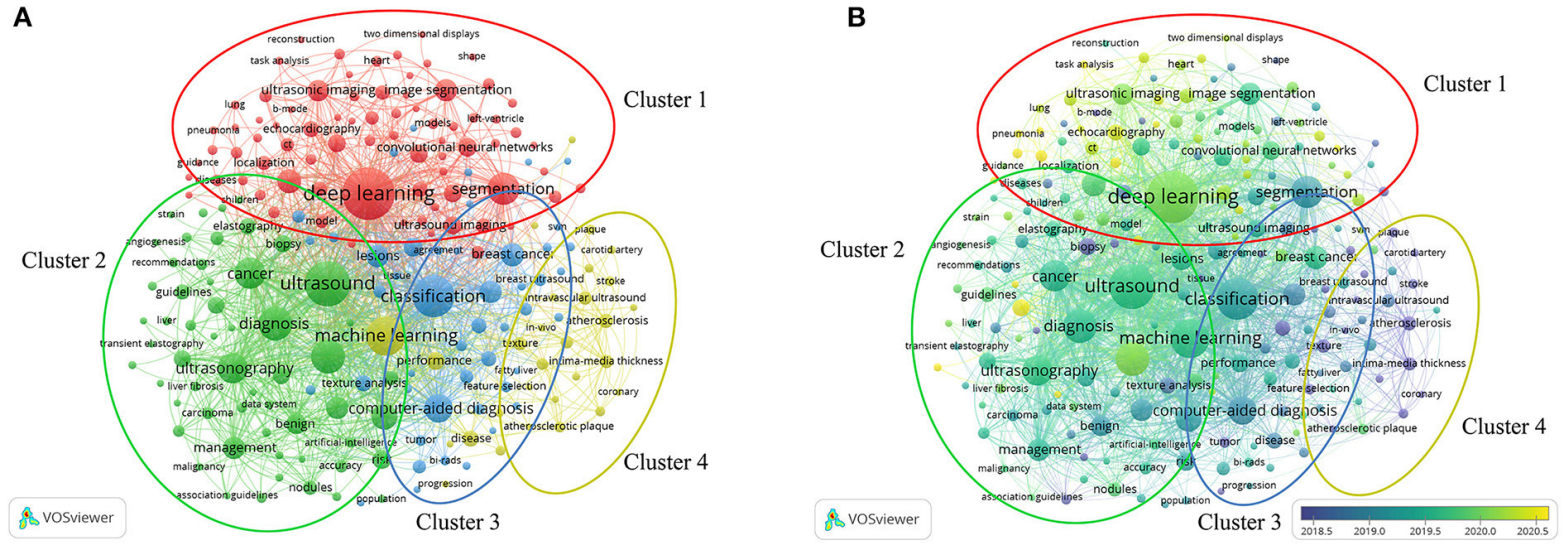
Co-occurrence analysis of all keywords in publications on the application of artificial intelligence in ultrasound. **(A)** Mapping the keywords of artificial intelligence applied in the field of ultrasound. The size of the circle indicates how often the keyword appears. **(B)** Present the distribution of keywords according to the average time of occurrence. The blue means earlier, and yellow means later.

Analyzing the results of this study by VOSview, we can intuitively get the latest keywords and the most frequent keywords. As shown in Figure 6, The co-occurrence analysis of keywords by VOSviewer visually presented four clusters which consisted of “deep learning,” “machine learning,” “application in the field of visceral organs,” and “application in the field of cardiovascular”. The latest hot word in cluster 1 (deep learning) was COVID-19 (Avg. pub. Year 2020.08). The latest hot word in cluster 2 (machine learning) was neural-network (Avg. pub. Year 2020.4). The latest hot word in the cluster 3 (applications in the field of visceral organs) was Hepatocellular Carcinoma (Avg. pub. Year 2020.6). The latest hot words in cluster 4 (application in the field of cardiovascular) was atherosclerotic plaques (Avg. pub. Year 2019.8).

### Dual-mapping overlay about AI in ultrasound

**Figure 7:**
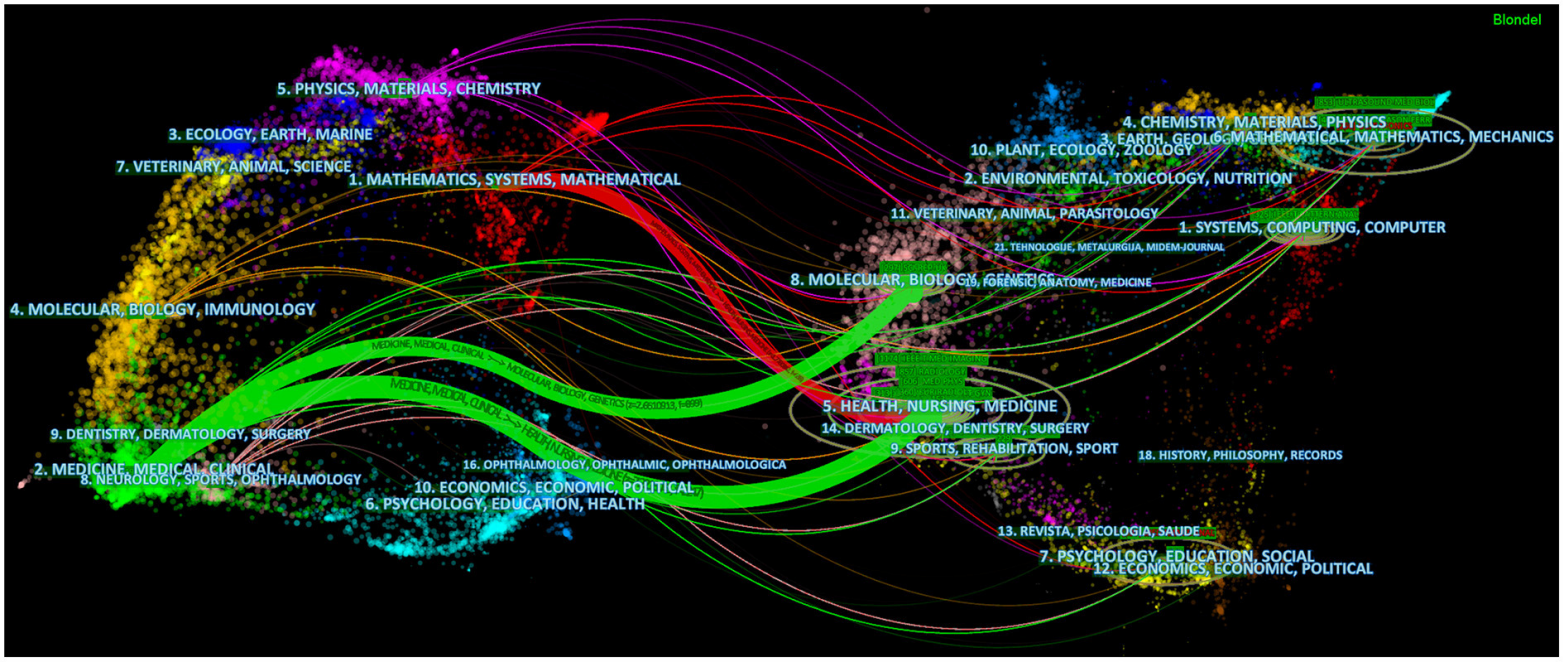
Dual-mapping overlay about AI applied in the field of ultrasound. The left side represents the fields of articles included in the study, and the right side represents the fields of references of articles.

The dual-mapping overlay reveals the overall scientific contributions. The left curve represents citing outline and the right curve represents cited outline. The curve is the quotation association line from the outside to the proper side, which explains the flow of knowledge and the connection between different research fields. Combined with this study, as shown in Figure 7, the left side of the green and red curves represents references cited in this study which mainly focus on medical, biology and mathematics and the right side of the curve represents the sources of references that literatures cite. This connection proves that publications we studied was closely related to mathematics and medical fields which conformed to the expected results of data processing mentioned above and keywords such as machine learning and ultrasound.

### Clinical trials about AI IN ultrasound

In order to further explore the interest of researchers in current clinical studies, we summarized the ongoing relevant clinical studies. The aim of this study is to demonstrate the relationship between the current clinical trials and the hot research directions. After manually excluding irrelevant search results, there were 5 results related to “artificial intelligence” and “ultrasound”. These experiments were conducted in the fields of endoscopic ultrasound, bedside ultrasound, echocardiography, breast ultrasound and lymph node classification of lung cancer. The method of study is mainly interventional and randomized. Two of the experiments were conducted in China. Four of the five studies were in recruitment mode, and one was completed in 2019 (Table 6).

**Table 6:** Clinical trials about AI in ultrasound.

**Study**	**ClinicalTrials.gov identifier**	**Office title**	**Country**	**Study type**	**No. of patients**	**Conditions**	**Intervention**	**Primary outcome measurement**	**Summary**
1	NCT04876157	Artificial Intelligence-aimed Point-of-care Ultrasound Image Interpretation System	China	Interventional	300	Ultrasound Image Interpretation	Diagnostic Test: Artificial intelligence-aimed point-of-care ultrasound image interpretation system	Sensitivity and specificity of AI interpretation	The main project is responsible for coordination between the two sub-projects and the main project, providing image resources, and using U-Net (Convolutional Networks for Biomedical Image Segmentation) and Transfer Learning to build up the models for image recognition and validating the efficacy of the models.
2	NCT05151939	Endoscopic Ultrasound (EUS) Artificial Intelligence Model for Normal Mediastinal and Abdominal Strictures Assessment	Ecuador	Observational	60	Abdomen; Mediastinum; Anatomic; Abnormality; Strictures	Diagnostic Test: Identification or discharge visualization of mediastinal and abdominal organ/anatomic strictures through Endoscopic ultrasound (EUS) videos by an expert endoscopist Diagnostic Test: Recognition of mediastinal and abdominal organ/anatomic strictures through Endoscopic ultrasound (EUS) videos using artificial intelligence (AI)	Overall accuracy of Endoscopic ultrasound (EUS) artificial intelligence (AI) model for identifying normal mediastinal and abdominal organ/anatomic strictures	Artificial intelligence (AI) aided recognition of anatomical structures may improve the training process and inter-observer agreement.
3	NCT04580095	Artificial Intelligence for Improved Echocardiography	Norway	Interventional	80	Heart Diseases	AI algorithm for apical foreshortening in echocardiography	Left ventricular apical foreshortening	The purpose of this study is to assess the effect of artificial intelligence algorithms on image quality in echocardiography.
4	NCT03849040	The Use of Artificial Intelligence to Predict Cancerous Lymph Nodes for Lung Cancer Staging During Ultrasound Imaging	Canada	Observational	52	Lung Diseases; Lung Neoplasm	Procedure: Endobronchial Ultrasound	Development of computer algorithm to identify lymph node ultrasonographic features Validation of computer algorithm to identify lymph node ultrasonographic features Accuracy and reliability of the segmentation performed by NeuralSeg NeuralSeg prediction of lymph node malignancy	This study aims to determine if a deep neural artificial intelligence (AI) network (NeuralSeg) can learn how to assign the Canada Lymph Node Score to lymph nodes examined by endobronchial ultrasound transbronchial needle aspiration (EBUS-TBNA), using the technique of segmentation.
5	NCT04270032	Using Deep Learning Methods to Analyze Automated Breast Ultrasound Images, to Establish a Diagnosis, Therapy Assessment and Prognosis Prediction Model of Breast Cancer.	China	Observational	10,000	Breast Cancer	Diagnostic Test: ABUS	Sensitivity false-positive per volume area under curve	The purpose of this study is using a deep learning method to analyze the automated breast ultrasound (ABUS) imagings, establish and evaluate a diagnosis, therapy assessment and prognosis prediction model of breast cancer.

## Discussion

**Figure 8:**
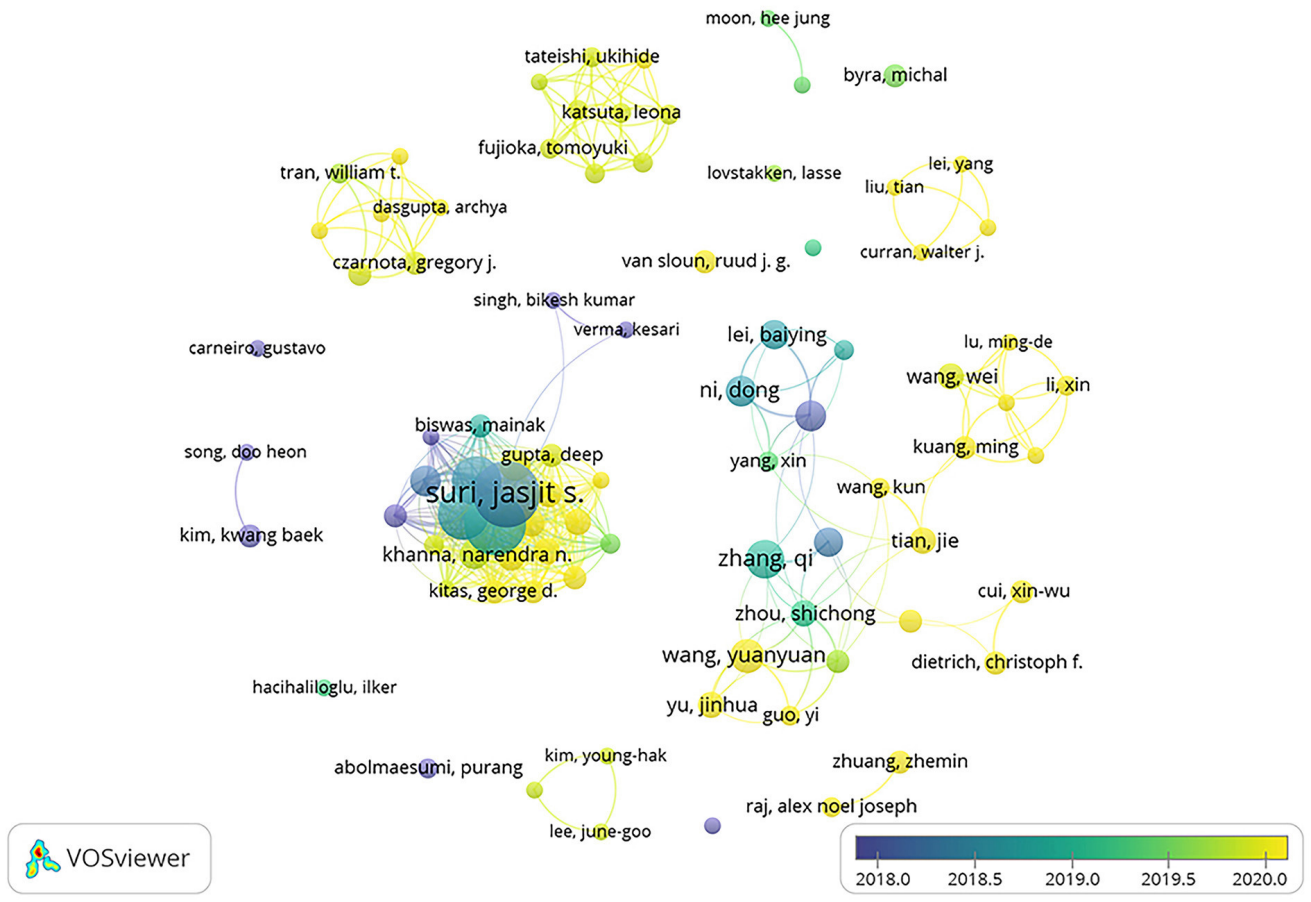
The network of top 10 authors produced in VOSviewer. The size of circles reveals the publications. The lines between the circles represent the connections between the authors.

**Figure 9:**
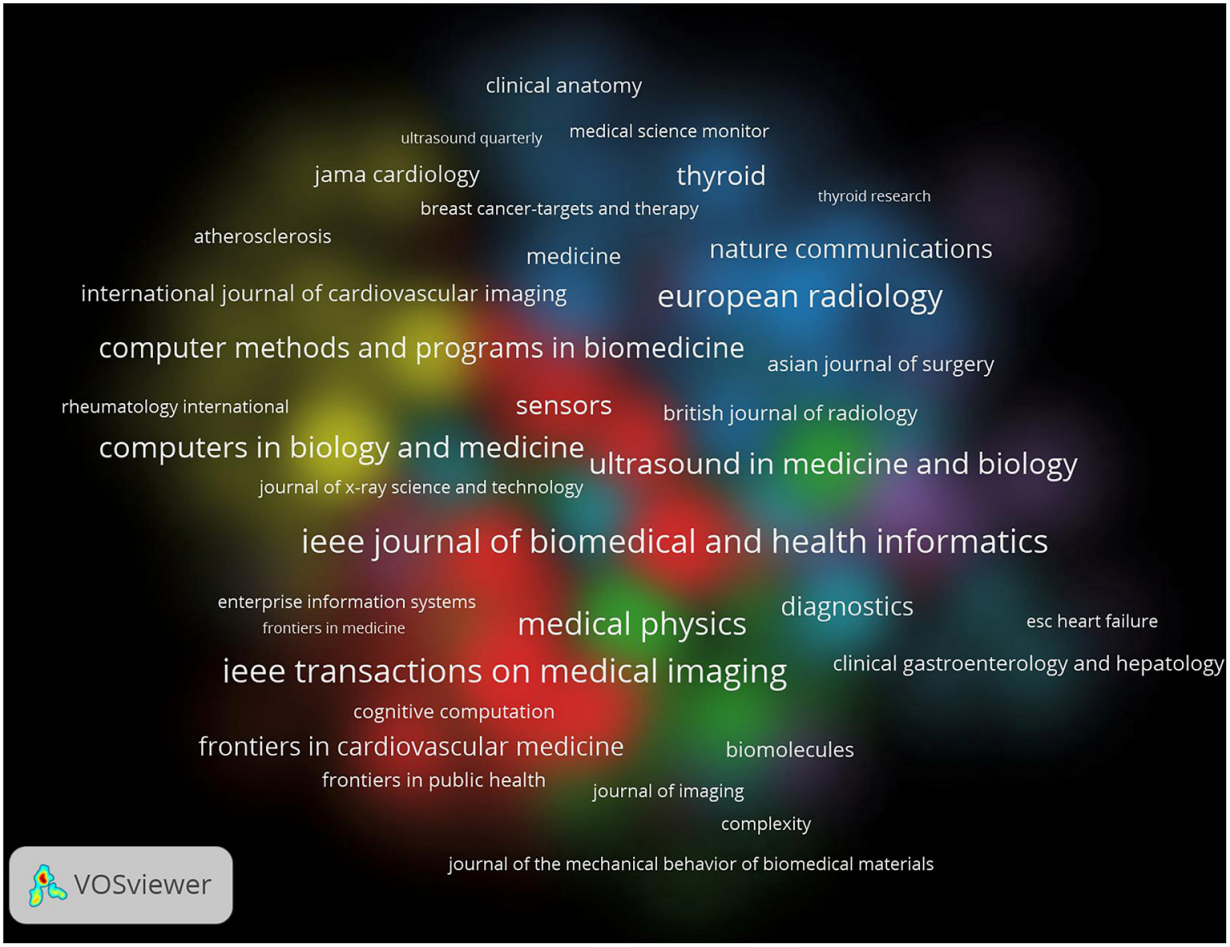
The network of journals produced in VOSviewer. The size of circles reveals the H-index.

China contributed most to the number of publications and the United States followed next. The development of AI technology is inseparable from the progress of machine learning technology and the progress of machine learning itself needs the support of a large amount of biological data as training materials, so we speculated that the emergence of this phenomenon was related to the large amount of biomedical data. China has a large population which provides a lot of training materials for DL algorithms. This could also explain why India was the third largest contributor. In recent years, the rise of big data has provided base with a large amount of data for DL training. The breakthrough of the fifth-generation communication technology has made it possible to transmit large amounts of data and information. Meanwhile, China has taken the AI industry as a vital development goal and put forward the “three-year plan for AI” and other measures since 2017. Therefore, we believe that the progress of science and technology and the support of national policies made China's contribution to the number of publications leading the world (10). The United States followed next as the second largest contributor. Since the United States is a developed country with advanced ultrasound technology, this result may relate to its strong economic strength. Countries with advanced productivity in the field of DL algorithms or ultrasound have better abilities to conduct research in this certain field. Moreover, according to the analysis by Vosviewer (Figure 8), the cooperation in this field between developed countries including the United States was very close and this cooperation played an important role in promoting the development of this field. Similar trends and logical analysis of publications were also reflected in the research on COVID-19, acute lung injury, heat stroke and sepsis (9, 11–13), which suggests that it is very important to strengthen the cooperation between multiple countries, institutions and scientific fields to promote the progress of scientific research effectively. Meanwhile, as shown in Figure 9 and Tables 2, 3, top journals in numbers of publications and times of citations were all found in the United States in the analysis of institutions and journals.

By analyzing the contribution of each country, it was found that China contributed the most to global publications. However, the US ranked first in our analysis of global publication contributions by journals and citations of publications, ahead of China. By analyzing the possible reasons for this phenomenon, it is our view that though the United States did not have large amounts of biomedical data, it could make up for the lack of data that required by DL training by optimizing the medical data algorithm. Meanwhile, according to our analysis in previous stage, developed countries led by the United States attached more importance to the cooperation between multiple fields and institutions, which can provide more exciting research hotspots and higher citations.

Among the top 10 authors with the most publications, 2 American authors and 3 Chinese authors had similar total numbers of publications, indicating that American authors had advantages in the number of publications. As shown in Table 4 and Figure 8, the total number of articles published by Chinese authors was similar to that of the United States, but it was more dispersed and gradually showed a trend of cooperation. Suri J. S., the author with the most publications, paid more attention to communication and cooperation, and showed a stronger cooperative relationship than other authors. Combined with the above analysis, it is further proved that multi-institution and multi-field cooperation can promote the progress of scientific research.

In our research, the highest co-citation was published in Medical Image Computing and Computer Assisted Intervention (14). This article mainly focused on the excellent performance of rolling neural network in the field of image analysis and the effective analysis of limited data could be realized through data enhancement. CNN is a deep learning algorithm that employs a hierarchical topology of connections inspired by the biological visual system, which allows it to learn the mapping of data features to categories (15). Recently, convolutional neural network has been applied more and more in the field of visual system and medical image analysis (16–18). A study showed that two different Convolutional Neural Networks could accurately classify 77.3 and 77.9% of the test atlas and the diagnostic accuracy of the test atlas was as high as 90.4 and 89.7%. Experienced radiologists analyzed 71.7% of the test atlas accurately. In contrast, the accuracy of Convolutional Neural Networks for image classification was slightly higher than that of human radiologists (15).

The top three most-cited articles, respectively, explained the value of deep learning in fetal location, breast disease and thyroid disease. In the first and second most-cited papers, it was emphasized that the transfer learning strategy could improve the methods of machine learning and the over-fitting problem caused by insufficient data was solved. The application and effectiveness of deep learning methods in image analysis and processing were confirmed in all three literatures which were also consistent with the hot status of AI applied to image analysis. As shown in Table 5, the IF of the top 10 literatures with the most citations in the field of application of AI assisted diagnosis in clinical practices were generally not high, and the academic interest in this field did not decrease (as shown in Figure 3A). Since 2011 (until 2021.06), the RRI index had been on an upward trend and witnessed a blowout performance recently. By our analysis, we believe that there are the following reasons that could explain this phenomenon: Firstly, the improvement of computer hardware, the emergence of 5G communication technology and the rise of big data technology in recent years provide advantages for the development of machine learning and deep learning. Secondly, the deep learning mode of roll neural network brings better image analysis and processing ability to the application of AI in the field of ultrasound (19, 20). Neural network technology was widely used in radiology and other fields of machine learning and Convolutional Neural Networks have been proved to have advantages in processing raw pixel data in the field of image classification. The speed, convenience and accuracy of AI-assisted ultrasound diagnosis are very valuable in clinical practices (21). For example, in the prevention and treatment of COVID-19 that swept the world in 2020, the appearance of AI-assisted diagnosis technology greatly reduced the ultrasonic diagnosis results' dependence on operators in some cases, which effectively shortened the manual interpretation time, reduced the cost of ultrasonic examination, improved the accuracy of results and significantly eased the strain on healthcare systems during the COVID-19 global pandemic (21, 22). Finally, different from CT or other invasive methods, ultrasound has advantages as a non-invasive and non-ionizing radiation method of examination, which makes its application in special groups such as pregnant women much safer. Ultrasound is also less sensitive to the influence of surrounding tissues compared with high-density imaging (DM) (21, 23, 24). We analyzed the research trend of AI application in ultrasonic field by keywords. As shown in Figure 6B, “COVID-19” was the latest keyword in publications. In recent years, the world has been affected by the SARS-CoV-2 and the diagnosis and management of patients with COVID-19 have become a hot research topic. Ultrasound plays an important role in the management of patients with COVID-19 because of its advantages of being non-invasive, portable, highly accurate and having bare impact on the control of infection. For example, a retrospective study in 2020 suggested that bedside ultrasound imaging of COVID-19 had advantages based on the characteristics of pathological progression of COVID-19 and the requirements on the management of moderate-to-severe patient.

The histopathological progression of COVID-19 began in the distal lung region and was characterized by alveolar damage and edema, thickening of lung interstitial and consolidation of lung tissue (25). Therefore, surface imaging techniques such as lung ultrasound were well suitable to observe the pathological progression of COVID-19 (26). As recommended by the World Health Organization, patients with moderate-to-severe COVID-19 need respiratory support and regular monitoring (27). For patients already receiving respiratory support, ultrasound can help monitor the progress of the diseases effectively (28). Bedside ultrasound which is more practical and radiation-free than chest radiography can be well suited for this situation. When facing great medical pressure, ultrasound can alleviate the dilemma of medical resources shortage and help control infection well (27). In addition, compared with other monitoring methods, pulmonary ultrasound is more competent in ergonomics and less in the possibility of infection (27, 29). Interestingly, according to the clinicaltrail database (https://www.clinicaltrials.gov/), the latest clinical trial uses artificial intelligence to analyze bronchial ultrasound images. The design was initiated in Canada using a prospective cohort study and validated by 100 images of lymph nodes. The purpose of the experiment was to explore whether CNN could realize lymph node classification through learning.

“Deep learning” was the keyword that appeared most frequently. According to relevant studies and our analysis, the possible reason for this situation is that breakthrough in big data sets, computer platforms and algorithms in recent years have led to significant progress in the field of AI in clinical medicine (30). Through machine learning, AI could improve its performance by learning numbers of data from the feedback of the algorithm. Machine learning, as a subdomain of AI, is to make predictions about unknown data by learning the inherent statistical patterns of large amounts of data. “Deep learning”, a machine learning technique based on artificial neural networks, is capable of learning and predicting complex data such as images through massive mathematical operations. AI technology has developed rapidly in recent years, relying on advances in computer hardware, the development of DL algorithms and large amounts of data training. With the application of convolutional neural network in image classification and the large amount of medical image data brought by transfer learning (31), DL has made great progress especially in image classification. The application of this technology in ultrasound and other clinical imageology can reduce the dependence on ultrasound doctors in certain degree, minimize the error caused by manual reading and make up for imaging doctors' lack of experience in some cases (1, 3). This undoubtedly brings great improvement to modern imageology, especially the “ultrasound technology” which is the object of this study. Over the past few decades, advances in high-throughput technology have significantly increased the amount of biomedical data. Algorithmic frameworks based on “deep learning” can handle, analyze and aggregate these intractable biomedical data (32). Constantly optimized DL algorithms, such as “transfer learning” can also relieve the dependence of “machine learning” on huge data volume in certain degree (15). Therefore, DL algorithm combined with convolutional neural network will continue being a research hotspot in next stage in the fields of ultrasonic diagnosis and technological innovation.

With the breakthroughs in AI and other fields and the advancement of multi-field integration, ultrasound, as a clinical examination tool, is bound to make continuous progress. Clinical trials always require the addition of more original technologies, so we have summarized the relevant content of clinical trials. Of the 5 relevant experiments, 2 were conducted in China, which is related to the country's emphasis on developing AI technology in recent years. It is worth noting that the research content of the ongoing clinical trials is consistent with the hot issues discussed above, such as echocardiography, breast ultrasound and pulmonary ultrasound. This result indicates that the application of AI combined with ultrasound in these hot areas has indeed aroused people's interest and can be effectively applied to the clinic. In addition, this result also tells us that the transition from theory to clinic is a critical step in solving clinical problems. After all, innovation theory needs practice to prove its validity (Table 6).

In addition, with the progress of technology, ultrasound imaging technology is more and more widely used in clinical practice. According to the known research results, ultrasound, as a non-invasive means, still has a good performance in the fields of adjuvant medication, such as the blood-brain barrier opening induced and the adjuvant drug delivery by ultrasound (33, 34). Combined with the rapid development of artificial intelligence technology in recent years, it is not difficult to see that ultrasound technology combined with artificial intelligence will make more breakthrough progress in the future. During this period, the continued strengthening of cooperation between major institutions and countries will certainly be an important part of promoting this historical process.

## Limitation

This study investigated publications from the WOS database from 2011 to 2021, and we tried to obtain objective and reliable results. However, due to the limitation of the search to studies in English and the constant updating of the database, as well as the exclusion of non-research articles, the results may differ slightly from the actual results. For more comprehensive results, databases such as Medline, Scopus, or Google Scholar could be searched in further studies.

## Conclusion

In conclusion, this paper summarizes and analyzes the global research trend of AI in imaging analysis and diagnosis assistance in the field of ultrasound. The number of global publications in this field has grown significantly in recent years and China and the United States have contributed the most in this field. While COVID-19 related research has become a hotspot of current research since world is being affected by a Coronavirus pandemic, deep learning algorithms included Convolutional Neural Networks will have remarkable development in this field in the future. In addition, combined with the results of this study, we predict that the research hot pot is the neural network-based DL algorithm and the transformation to clinical applications. Our results provide profound insights into the current status and history of this field, and predict its development trend.

## Data availability statement

The original contributions presented in the study are included in the article/Supplementary material, further inquiries can be directed to the corresponding author/s.

## Author contributions

Conception and design: YW. Provision of study materials or patients: KW. Collection and assembly of data, data analysis, and interpretation: DX, GC, KW, and MY. Manuscript writing and final approval of manuscript: all authors.

## Funding

This study was supported by 2019 Technical Standard Project of Shanghai Science and Technology Innovation Action Plan of Science and Technology Commission of Shanghai Municipality (19DZ2203300) and Clinical Research Foundation of Shanghai Pulmonary Hospital (Fk1940, FKLY20015).

## Conflict of interest

The authors declare that the research was conducted in the absence of any commercial or financial relationships that could be construed as a potential conflict of interest.

## Publisher's note

All claims expressed in this article are solely those of the authors and do not necessarily represent those of their affiliated organizations, or those of the publisher, the editors and the reviewers. Any product that may be evaluated in this article, or claim that may be made by its manufacturer, is not guaranteed or endorsed by the publisher.
