# Supplementary material for: Research progress and hotspot of the artificial intelligence application in the ultrasound during 2011–2021: A bibliometric analysis
Source: Front Public Health. 2022 Sep 15;10:990708. doi: 10.3389/fpubh.2022.990708 (PMC9520910; doi:10.3389/fpubh.2022.990708)
Supplement: Supplementary file 1 [file Table_1.DOCX]

**Supplementary material**

| id | label | cluster | weight<Links> | weight<Total link strength> | weight<Occurrences> | score<Avg. pub. year> |
| --- | --- | --- | --- | --- | --- | --- |
| 1 | covid-19 | 1 | 36 | 74 | 15 | 2020.8 |
| 2 | pneumonia | 1 | 33 | 51 | 8 | 2020.714 |
| 3 | magnetic resonance imaging | 1 | 45 | 72 | 13 | 2020.667 |
| 4 | lung ultrasound | 1 | 27 | 34 | 11 | 2020.546 |
| 5 | image classification | 1 | 28 | 38 | 7 | 2020.5 |
| 6 | task analysis | 1 | 41 | 69 | 8 | 2020.5 |
| 7 | force | 1 | 22 | 27 | 7 | 2020.429 |
| 8 | tomography | 1 | 29 | 34 | 6 | 2020.4 |
| 9 | tumor segmentation | 1 | 20 | 27 | 6 | 2020.4 |
| 10 | brain | 1 | 32 | 37 | 8 | 2020.375 |
| 11 | echocardiography | 1 | 55 | 119 | 29 | 2020.345 |
| 12 | acoustics | 1 | 48 | 78 | 12 | 2020.333 |
| 13 | attention mechanism | 1 | 18 | 26 | 7 | 2020.333 |
| 14 | brachytherapy | 1 | 22 | 33 | 9 | 2020.333 |
| 15 | biomedical imaging | 1 | 55 | 106 | 13 | 2020.308 |
| 16 | semantic segmentation | 1 | 36 | 60 | 13 | 2020.308 |
| 17 | convolutional neural-network | 1 | 34 | 53 | 8 | 2020.286 |
| 18 | training | 1 | 65 | 138 | 19 | 2020.263 |
| 19 | ct | 1 | 64 | 126 | 21 | 2020.25 |
| 20 | data augmentation | 1 | 27 | 33 | 8 | 2020.25 |
| 21 | deep | 1 | 38 | 48 | 8 | 2020.25 |
| 22 | lung | 1 | 36 | 65 | 9 | 2020.222 |
| 23 | medical imaging | 1 | 41 | 59 | 10 | 2020.2 |
| 24 | imaging | 1 | 70 | 134 | 23 | 2020.182 |
| 25 | cnn | 1 | 64 | 115 | 17 | 2020.177 |
| 26 | generative adversarial network | 1 | 25 | 32 | 6 | 2020.167 |
| 27 | point-of-care ultrasound | 1 | 14 | 20 | 6 | 2020.167 |
| 28 | surgery | 1 | 21 | 24 | 6 | 2020.167 |
| 29 | two dimensional displays | 1 | 24 | 37 | 6 | 2020.167 |
| 30 | mri | 1 | 89 | 189 | 28 | 2020.148 |
| 31 | heart | 1 | 46 | 73 | 14 | 2020.143 |
| 32 | tracking | 1 | 53 | 94 | 20 | 2020.05 |
| 33 | ultrasonic imaging | 1 | 113 | 344 | 50 | 2020.02 |
| 34 | convolutional neural-networks | 1 | 59 | 126 | 18 | 2020 |
| 35 | deep learning | 1 | 199 | 1508 | 305 | 2020 |
| 36 | guidance | 1 | 12 | 15 | 6 | 2020 |
| 37 | left-ventricle | 1 | 35 | 52 | 9 | 2020 |
| 38 | neural network | 1 | 53 | 91 | 15 | 2020 |
| 39 | u-net | 1 | 51 | 80 | 16 | 2020 |
| 40 | pregnancy | 1 | 27 | 41 | 10 | 2019.9 |
| 41 | networks | 1 | 47 | 69 | 12 | 2019.833 |
| 42 | convolutional neural network | 1 | 108 | 291 | 64 | 2019.8 |
| 43 | convolutional neural networks | 1 | 82 | 194 | 39 | 2019.757 |
| 44 | computed tomography | 1 | 42 | 66 | 9 | 2019.714 |
| 45 | models | 1 | 42 | 73 | 14 | 2019.714 |
| 46 | feature extraction | 1 | 95 | 246 | 35 | 2019.677 |
| 47 | localization | 1 | 58 | 118 | 24 | 2019.667 |
| 48 | image segmentation | 1 | 113 | 290 | 56 | 2019.618 |
| 49 | convolutional neural network (cnn) | 1 | 48 | 70 | 13 | 2019.615 |
| 50 | transfer learning | 1 | 86 | 214 | 37 | 2019.611 |
| 51 | breast imaging | 1 | 22 | 37 | 7 | 2019.571 |
| 52 | detection | 1 | 39 | 56 | 7 | 2019.571 |
| 53 | diseases | 1 | 58 | 114 | 15 | 2019.571 |
| 54 | reconstruction | 1 | 30 | 34 | 7 | 2019.571 |
| 55 | network | 1 | 48 | 73 | 14 | 2019.539 |
| 56 | breast tumors | 1 | 33 | 45 | 6 | 2019.5 |
| 57 | enhancement | 1 | 41 | 58 | 9 | 2019.5 |
| 58 | ultrasound imaging | 1 | 95 | 205 | 38 | 2019.447 |
| 59 | model | 1 | 59 | 95 | 18 | 2019.375 |
| 60 | ultrasound image | 1 | 83 | 164 | 35 | 2019.281 |
| 61 | reliability | 1 | 49 | 64 | 11 | 2019.273 |
| 62 | selection | 1 | 31 | 57 | 8 | 2019.25 |
| 63 | speckle | 1 | 32 | 46 | 9 | 2019.222 |
| 64 | volume | 1 | 23 | 32 | 7 | 2019.143 |
| 65 | segmentation | 1 | 152 | 667 | 116 | 2019.099 |
| 66 | fetal ultrasound | 1 | 10 | 16 | 6 | 2019 |
| 67 | neural-networks | 1 | 79 | 142 | 28 | 2018.885 |
| 68 | neural networks | 1 | 27 | 41 | 8 | 2018.875 |
| 69 | prostate | 1 | 24 | 31 | 6 | 2018.833 |
| 70 | framework | 1 | 53 | 66 | 12 | 2018.818 |
| 71 | quantification | 1 | 80 | 142 | 23 | 2018.739 |
| 72 | prostate cancer | 1 | 33 | 56 | 12 | 2018.727 |
| 73 | shape | 1 | 25 | 30 | 6 | 2018.667 |
| 74 | 3d ultrasound | 1 | 21 | 23 | 7 | 2018.6 |
| 75 | thickness | 1 | 19 | 28 | 7 | 2018.571 |
| 76 | b-mode | 1 | 31 | 40 | 6 | 2018.333 |
| 77 | automatic segmentation | 1 | 36 | 47 | 11 | 2018.091 |
| 78 | hepatocellular carcinoma | 2 | 22 | 34 | 8 | 2020.625 |
| 79 | diagnostic imaging | 2 | 22 | 32 | 6 | 2020.6 |
| 80 | artificial intelligence (ai) | 2 | 21 | 24 | 7 | 2020.5 |
| 81 | radiomics | 2 | 81 | 198 | 33 | 2020.433 |
| 82 | risk stratification | 2 | 57 | 90 | 10 | 2020.3 |
| 83 | doppler | 2 | 37 | 51 | 10 | 2020.1 |
| 84 | artificial intelligence | 2 | 160 | 752 | 129 | 2020.05 |
| 85 | elasticity imaging techniques | 2 | 31 | 44 | 6 | 2020 |
| 86 | hepatocellular-carcinoma | 2 | 38 | 54 | 7 | 2020 |
| 87 | liver fibrosis | 2 | 40 | 67 | 11 | 2020 |
| 88 | strain | 2 | 24 | 35 | 7 | 2020 |
| 89 | data system | 2 | 51 | 92 | 10 | 2019.9 |
| 90 | ultrasound elastography | 2 | 42 | 61 | 9 | 2019.889 |
| 91 | guidelines | 2 | 77 | 170 | 26 | 2019.84 |
| 92 | angiogenesis | 2 | 27 | 37 | 6 | 2019.833 |
| 93 | association guidelines | 2 | 28 | 51 | 6 | 2019.833 |
| 94 | nodules | 2 | 63 | 157 | 24 | 2019.826 |
| 95 | prediction | 2 | 70 | 134 | 24 | 2019.818 |
| 96 | malignancy | 2 | 39 | 73 | 8 | 2019.75 |
| 97 | artificial-intelligence | 2 | 44 | 79 | 12 | 2019.727 |
| 98 | white paper | 2 | 29 | 43 | 7 | 2019.714 |
| 99 | ultrasonography | 2 | 126 | 592 | 101 | 2019.68 |
| 100 | ultrasound | 2 | 194 | 1294 | 231 | 2019.671 |
| 101 | shear-wave elastography | 2 | 60 | 105 | 12 | 2019.667 |
| 102 | elastography | 2 | 87 | 182 | 29 | 2019.571 |
| 103 | random forest | 2 | 33 | 48 | 7 | 2019.571 |
| 104 | thyroid | 2 | 40 | 66 | 8 | 2019.571 |
| 105 | thyroid nodule | 2 | 77 | 246 | 34 | 2019.559 |
| 106 | diagnosis | 2 | 166 | 770 | 127 | 2019.542 |
| 107 | thyroid cancer | 2 | 49 | 118 | 15 | 2019.533 |
| 108 | accuracy | 2 | 78 | 143 | 19 | 2019.529 |
| 109 | cancer | 2 | 146 | 764 | 109 | 2019.51 |
| 110 | liver | 2 | 38 | 56 | 9 | 2019.5 |
| 111 | recommendations | 2 | 54 | 84 | 12 | 2019.5 |
| 112 | variability | 2 | 32 | 39 | 6 | 2019.5 |
| 113 | management | 2 | 94 | 427 | 58 | 2019.464 |
| 114 | society | 2 | 34 | 50 | 8 | 2019.429 |
| 115 | us | 2 | 95 | 285 | 33 | 2019.394 |
| 116 | shear wave elastography | 2 | 33 | 47 | 6 | 2019.333 |
| 117 | differentiation | 2 | 44 | 76 | 10 | 2019.3 |
| 118 | children | 2 | 20 | 29 | 8 | 2019.286 |
| 119 | thyroid nodules | 2 | 54 | 157 | 20 | 2019.263 |
| 120 | risk | 2 | 105 | 280 | 41 | 2019.205 |
| 121 | cirrhosis | 2 | 47 | 87 | 12 | 2019.2 |
| 122 | contrast-enhanced ultrasound | 2 | 50 | 85 | 16 | 2019.2 |
| 123 | transient elastography | 2 | 25 | 40 | 6 | 2019.2 |
| 124 | features | 2 | 136 | 538 | 75 | 2019.183 |
| 125 | carcinoma | 2 | 48 | 109 | 15 | 2019.143 |
| 126 | population | 2 | 41 | 62 | 8 | 2019.125 |
| 127 | system | 2 | 122 | 347 | 55 | 2019.082 |
| 128 | benign | 2 | 103 | 377 | 48 | 2019.063 |
| 129 | differential-diagnosis | 2 | 52 | 99 | 14 | 2018.923 |
| 130 | fibrosis | 2 | 44 | 66 | 10 | 2018.9 |
| 131 | fine-needle-aspiration | 2 | 40 | 89 | 11 | 2018.546 |
| 132 | criteria | 2 | 37 | 48 | 7 | 2018.429 |
| 133 | biopsy | 2 | 94 | 196 | 32 | 2018.3 |
| 134 | lesion classification | 2 | 42 | 68 | 8 | 2018 |
| 135 | neural-network | 3 | 46 | 65 | 11 | 2020.4 |
| 136 | computer-aided diagnosis (cad) | 3 | 30 | 45 | 7 | 2020.167 |
| 137 | svm | 3 | 22 | 26 | 6 | 2020.167 |
| 138 | breast tumor | 3 | 25 | 34 | 6 | 2020 |
| 139 | cancer statistics | 3 | 23 | 30 | 6 | 2020 |
| 140 | deep neural networks | 3 | 22 | 31 | 7 | 2020 |
| 141 | interobserver variability | 3 | 27 | 35 | 6 | 2020 |
| 142 | breast | 3 | 60 | 146 | 19 | 2019.947 |
| 143 | cancer detection | 3 | 37 | 53 | 10 | 2019.889 |
| 144 | image processing | 3 | 19 | 26 | 6 | 2019.833 |
| 145 | breast cancer | 3 | 109 | 358 | 62 | 2019.632 |
| 146 | lesions | 3 | 119 | 398 | 57 | 2019.554 |
| 147 | women | 3 | 54 | 136 | 21 | 2019.476 |
| 148 | bi-rads | 3 | 45 | 82 | 11 | 2019.455 |
| 149 | tumors | 3 | 55 | 110 | 19 | 2019.368 |
| 150 | agreement | 3 | 38 | 65 | 9 | 2019.333 |
| 151 | lesion detection | 3 | 20 | 27 | 6 | 2019.333 |
| 152 | mammography | 3 | 71 | 193 | 28 | 2019.321 |
| 153 | identification | 3 | 32 | 43 | 8 | 2019.286 |
| 154 | quantitative ultrasound | 3 | 25 | 38 | 11 | 2019.273 |
| 155 | support vector machine | 3 | 60 | 106 | 20 | 2019.263 |
| 156 | breast ultrasound | 3 | 61 | 110 | 17 | 2019.25 |
| 157 | ultrasound images | 3 | 89 | 217 | 40 | 2019.235 |
| 158 | classification | 3 | 191 | 1234 | 194 | 2019.22 |
| 159 | computer-aided detection | 3 | 40 | 66 | 10 | 2019.2 |
| 160 | artificial neural network | 3 | 22 | 28 | 6 | 2019.167 |
| 161 | information | 3 | 40 | 55 | 8 | 2019.125 |
| 162 | computer-aided diagnosis | 3 | 139 | 643 | 92 | 2019 |
| 163 | texture features | 3 | 31 | 41 | 8 | 2019 |
| 164 | images | 3 | 124 | 353 | 58 | 2018.912 |
| 165 | recognition | 3 | 26 | 45 | 9 | 2018.889 |
| 166 | machine | 3 | 41 | 52 | 9 | 2018.875 |
| 167 | sonography | 3 | 52 | 100 | 14 | 2018.857 |
| 168 | fatty liver | 3 | 33 | 43 | 6 | 2018.833 |
| 169 | prevalence | 3 | 44 | 72 | 13 | 2018.833 |
| 170 | masses | 3 | 70 | 148 | 19 | 2018.79 |
| 171 | texture analysis | 3 | 69 | 157 | 29 | 2018.724 |
| 172 | feature-selection | 3 | 55 | 104 | 14 | 2018.539 |
| 173 | impact | 3 | 32 | 38 | 8 | 2018.5 |
| 174 | tissue | 3 | 38 | 52 | 8 | 2018.375 |
| 175 | computer aided diagnosis | 3 | 45 | 63 | 9 | 2018.25 |
| 176 | breast-cancer | 3 | 27 | 34 | 6 | 2018.167 |
| 177 | algorithm | 3 | 84 | 151 | 25 | 2018.083 |
| 178 | medical image processing | 3 | 36 | 46 | 8 | 2018 |
| 179 | tumor | 3 | 50 | 92 | 13 | 2017.9 |
| 180 | feature selection | 3 | 29 | 32 | 6 | 2017.833 |
| 181 | support vector machines | 3 | 47 | 70 | 10 | 2017.7 |
| 182 | stratification | 4 | 32 | 49 | 7 | 2020.5 |
| 183 | diagnostic-accuracy | 4 | 35 | 48 | 10 | 2020.1 |
| 184 | atherosclerotic plaque | 4 | 47 | 105 | 13 | 2019.846 |
| 185 | risk assessment | 4 | 37 | 56 | 8 | 2019.75 |
| 186 | machine learning | 4 | 184 | 935 | 171 | 2019.652 |
| 187 | computed-tomography | 4 | 27 | 37 | 6 | 2019.5 |
| 188 | performance | 4 | 113 | 303 | 36 | 2019.389 |
| 189 | lumen diameter | 4 | 23 | 43 | 6 | 2019.333 |
| 190 | prognosis | 4 | 11 | 14 | 6 | 2019.333 |
| 191 | stenosis | 4 | 40 | 70 | 7 | 2019.333 |
| 192 | progression | 4 | 24 | 29 | 6 | 2019.167 |
| 193 | magnetic-resonance | 4 | 36 | 49 | 7 | 2019.143 |
| 194 | ivus | 4 | 32 | 48 | 9 | 2019.111 |
| 195 | tissue characterization | 4 | 67 | 144 | 18 | 2019.056 |
| 196 | association | 4 | 49 | 73 | 11 | 2018.9 |
| 197 | disease | 4 | 72 | 151 | 30 | 2018.897 |
| 198 | validation | 4 | 62 | 117 | 16 | 2018.867 |
| 199 | intravascular ultrasound | 4 | 46 | 90 | 22 | 2018.818 |
| 200 | lumen | 4 | 15 | 25 | 6 | 2018.667 |
| 201 | stroke | 4 | 37 | 73 | 11 | 2018.546 |
| 202 | accurate | 4 | 39 | 56 | 7 | 2018.429 |
| 203 | imt measurement | 4 | 31 | 85 | 12 | 2018.273 |
| 204 | texture | 4 | 71 | 155 | 21 | 2018.191 |
| 205 | in-vivo | 4 | 28 | 32 | 6 | 2018.167 |
| 206 | coronary | 4 | 25 | 39 | 6 | 2018 |
| 207 | atherosclerosis | 4 | 56 | 172 | 24 | 2017.913 |
| 208 | b-mode ultrasound | 4 | 36 | 45 | 7 | 2017.857 |
| 209 | intima-media thickness | 4 | 47 | 156 | 22 | 2017.762 |
| 210 | plaque | 4 | 44 | 76 | 11 | 2017.273 |
| 211 | wall | 4 | 28 | 46 | 8 | 2016.125 |
| 212 | carotid artery | 4 | 30 | 45 | 8 | 2015.5 |
